# Efficient Particle Aggregation Through SSAW Phase Modulation

**DOI:** 10.3390/mi16080910

**Published:** 2025-08-05

**Authors:** Yiming Li, Zekai Li, Zuozhi Wei, Yiran Wang, Xudong Niu, Dongfang Liang

**Affiliations:** 1 Department of Engineering, University of Cambridge, Cambridge CB2 1PZ, UK; yl842@cam.ac.uk (Y.L.);; 2 South China National Center of Metrology, Guangdong Institute of Metrology, Guangzhou 510405, China; 3 College of Food Science and Engineering, Tianjin University of Science & Technology, Tianjin 300457, China; 4 Changchun Institute of Optics, Fine Mechanics and Physics, Chinese Academy of Sciences, Changchun 130033, China

**Keywords:** microfluidics, acoustofluidics, surface acoustic waves, particle manipulation

## Abstract

In recent years, various devices utilizing surface acoustic waves (SAW) have emerged as powerful tools for manipulating particles and fluids in microchannels. Although they demonstrate a wide range of functionalities across diverse applications, existing devices still face limitations in flexibility, manipulation efficiency, and spatial resolution. In this study, we developed a dual-sided standing surface acoustic wave (SSAW) device that simultaneously excites acoustic waves through two piezoelectric substrates positioned at the top and bottom of a microchannel. By fully exploiting the degrees of freedom offered by two pairs of interdigital transducers (IDTs) on each substrate, the system enables highly flexible control of microparticles. To explore its capability on particle aggregation, we developed a two-dimensional numerical model to investigate the influence of the SAW phase modulation on the established acoustic fields within the microchannel. Single-particle motion was first examined under the influence of the phase-modulated acoustic fields to form a reference for identifying effective phase modulation strategies. Key parameters, such as the phase changes and the duration of each phase modulation step, were determined to maximize the lateral motion while minimizing undesired vertical motion of the particle. Our dual-sided SSAW configuration, combined with novel dynamic phase modulation strategy, leads to rapid and precise aggregation of microparticles towards a single focal point. This study sheds new light on the design of acoustofluidic devices for efficient spatiotemporal particle concentration.

## 1. Introduction

Acoustofluidic techniques have emerged as powerful tools for the contactless manipulation of nano- and microscale particles in microfluidic systems [1,2,3]. By using sound waves to generate forces in fluids, these methods can precisely control fluid flow and particle motion without physical contact, offering excellent biocompatibility and minimal damage to cells [4,5,6,7,8]. In particular, surface acoustic wave (SAW) devices have gained prominence due to their simple planar geometry, precise microfabricated dimensions, and easy integration with microchannels [9,10,11,12]. A standing SAW (SSAW) field is typically established by a pair of opposing interdigital transducers (IDTs) on a piezoelectric substrate, which generates an acoustic resonance with pressure nodes (PNs) and antinodes (ANs) in the fluid chamber. Suspended particles within the fluid cavity experience an acoustic radiation force that drives them toward these pressure nodal planes, causing the particles to accumulate at the nodes. This approach has been widely used for tasks such as particle focusing [13,14,15], separation [16,17,18,19], and patterning [20,21,22] in microfluidics, enabling label-free and non-invasive handling of biological samples [23,24,25,26]. This acoustically induced, versatile manipulation of bioparticles in microfluidic systems is commonly referred to as an “acoustic tweezer,” serving as a powerful and promising tool for a wide range of lab-on-a-chip applications in engineering, biology, and chemistry.

Conventional single-SSAW microfluidic devices, which feature a pair of IDTs on the bottom substrate, have inherent limitations in aggregation efficiency. In such devices, particles typically collect along an extended nodal line across the microchannel (either the pressure nodal plane for particles with a positive contrast factor or the antinodal plane for those with a negative contrast factor), driven by the acoustic radiation force. Once particles reach nodal planes, their lateral migration becomes extremely slow due to small driving force, which significantly hampers the speed of particle concentration and consequently introduces multiple collection areas within a wavelength width [9]. Furthermore, with only one pair of transducers driving the acoustic field from a single side (usually from the substrate bottom), the acoustic energy input is limited [27]. This constraint results in a lower acoustic pressure amplitude in the fluid and hence slower particle transport velocities toward the nodes. Moreover, traditional acoustic fields tend to drive particles toward the microchannel boundaries, often leading to undesirable particle accumulation and clogging near the walls. As a consequence, aggregation of particles into a dense cluster can be inefficient in single-SSAW setups. One strategy to address this issue is to dynamically shift the phase of the acoustic field. For example, Orloff et al. [28] demonstrated that varying the relative electronic phase between two opposing IDTs can translate the positions of pressure nodes, thereby actively moving particles to designated locations. While such phase-controlled adjustments make it possible to drive particles along the channel width, in-plane particle manipulation is still relatively slow and lacks flexibility (particle motions refined to one dimension). Given that effective particle aggregation requires shuttling particles along the entire length of the pressure nodal line, phase modulation in a single-SSAW device remains insufficient to achieve this efficiently.

To overcome these limitations, many researchers have explored acoustofluidic designs with more than a pair of IDTs to generate dual-SSAW fields or multi-SSAW fields on the bottom substrate, offering new degrees of control over the pressure node pattern through interference of multiple SSAWs. For instance, Ding et al. [26] used dual-SSAW field established by two orthogonally oriented SSAW fields to reconfigure the PNs in a static fluid, enabling flexible and precise manipulation of cells and organisms. By introducing more IDTs and leveraging the anisotropic nature of piezoelectric substrate, Tian et al. [8] introduced wave number–spiral acoustic tweezers, which employ multitone and multi-angle SAW IDT arrays to dynamically reshape pressure fields, enabling programmable and precise reconfiguration of particle and cell patterns. Extending to three-dimensional manipulation, Ren et al. [6] developed a SSAW-based platform using joint subarray IDTs to manipulate cells in multiple degrees of freedom—including translation, rotation, and deformation—by dynamically tuning the phase and amplitude of the acoustic fields. These approaches demonstrated high flexibility and spatial resolution in single-cell manipulation. While these multi-SSAW devices configured on one side of the fluid cavity (typically the bottom side of the fluid cavity) offer strong potential for effective in-plane manipulation, achieving flexible out-of-plane manipulation remains challenging. This limitation arises because the standing wave pattern is confined to the plane parallel to the substrate surface, and phase modulation of in-plane SAWs does not effectively alter the pressure nodal planes in the out-of-plane direction.

Therefore, in our study, we propose a dual-sided SSAW device with two pairs of IDTs arranged to sandwich the microfluidic channel, which can produce acoustic waves from two sides (i.e., from the top and bottom boundaries of the fluid cavity), simultaneously forming a two-dimensional standing wave pattern in both the in-plane and out-of-plane directions. This setup allows more acoustic energy to be introduced into the fluid, thereby enhancing the efficiency of particle manipulation. Moreover, the dual-sided SSAW platform supports dynamic phase modulation of the acoustic field, enabling controlled out-of-plane particle manipulation, which typically relies on gravitational effects in conventional single-sided SSAW setups. By adjusting the relative phases of SAWs generated from the four IDTs, one can redistribute the acoustic pressure landscape in real time, effectively imparting additional momentum to particles along the nodal plane. A suitable phase difference between four SAWs can speed up their convergence and swiftly push them towards a single focal aggregation point. This phase modulation approach overcomes the slow lateral transport in a static SSAW by actively conveying particles towards the only aggregation point.

To the best of the author’s knowledge, this is the first time SSAW fields have been patterned from two sides for phase-modulated particle manipulation. Therefore, in the following sections, we detail the theoretical framework and design of this dual-sided SSAW acoustofluidic device while presenting numerical analysis of the acoustic pressure field and particle trajectories under multiple phase modulation methods using the finite element method (FEM). Rationales behind the selection of key parameters (i.e., phase difference, duration of acoustic treatment, etc.) are also explained in detail. The numerical results indicate that our dynamic phase modulation methodology utilized in a dual-sided SSAW device successfully achieves particle aggregation at single collection point within a minute. Our studies provide new insights into dynamic SSAW field patterning and acoustofluidic chip design for particle manipulation.

## 2. Computational Methods

### 2.1. Mathematical Models

#### 2.1.1. Governing Equations for Fluid Dynamics

In our model, the fluid is considered to be compressible and viscous, and therefore is governed by the conservation of mass and momentum, given as:

(1) $$\frac{\partial \rho }{\partial t}+\nabla \cdot \left(\rho v\right)=0$$

(2) $$\rho \frac{\partial v}{\partial t}+\rho \left(v\cdot \nabla \right)v=-\nabla p+\mu \nabla^{2}v+\left(\mu_{b}+\frac{\mu }{3}\right)\nabla \left(\nabla \cdot v\right)$$

where *ρ*, v, p, μ, and $\mu_{b}$ are the mass density distribution, fluid velocity, fluid pressure, shear viscosity, and bulk viscosity, respectively. To close the system, a linearized isentropic equation of state $p_{1}=c_{0}^{2}\rho_{1}$ is applied, with c_0_ being the isentropic speed of sound in the fluid. The challenge in solving Equations (1) and (2) arises from the scale gap between MHz-range acoustic oscillations and much slower acoustic streaming. Therefore, the acoustofluidic fields are expanded as perturbation series to the second order:

(3a) $$v=v_{0}+v_{1}+v_{2}+O\left(\epsilon^{3}\right)+\cdots$$

(3b) $$p=p_{0}+p_{1}+p_{2}+O\left(\epsilon^{3}\right)+\cdots$$

(3c) $$\rho =\rho_{0}+\rho_{1}+\rho_{2}+O\left(\epsilon^{3}\right)+\cdots$$

where the subscript 0 refers to the hydrostatic zeroth-order component, 1 refers to the harmonic first-order component, and 2 refers to the second-order streaming component. Substituting Equation (3) into Equations (1) and (2) and retaining all the first-order terms results in the following first-order equations:

(4) $$\frac{\partial \rho_{1}}{\partial t}=-\rho_{0}\nabla \cdot v_{1}$$

(5) $$\rho_{0}\frac{\partial v_{1}}{\partial t}=-\nabla p_{1}+\mu \nabla^{2}v_{1}+\left(\mu_{b}+\frac{\mu }{3}\right)\nabla (\nabla \cdot v_{1})$$

Time-averaging the second-order terms over a period of acoustic vibration T yields the following second-order streaming equations:

(6) $$-\nabla \cdot \langle \rho_{1}\cdot v_{1}\rangle =\rho_{0}\nabla \cdot \langle v_{2}\rangle$$

(7) $$\langle \rho_{1}\frac{\partial v_{1}}{\partial t}\rangle +\rho_{0}\langle (v_{1}\cdot \nabla )v_{1}\rangle =-\nabla \cdot \langle p_{2}\rangle +\mu \nabla^{2}\langle v_{2}\rangle +\left(\mu_{b}+\frac{\mu }{3}\right)\nabla \left(\nabla \cdot \langle v_{2}\rangle \right)$$

where   denotes temporal averaging, the resulting steady velocity $\langle v_{2}\rangle$ represents acoustic streaming, and $\langle p_{2}\rangle$ contributes to acoustic radiation force.

#### 2.1.2. Acoustophoretic Particle Manipulation

The forces acting on microparticles include the acoustic radiation force, viscous drag force, gravity, and buoyancy. Assuming neutrally buoyant particles in dilute suspensions with radii much smaller than the acoustic wavelength, we can neglect both particle–particle interactions and particle-induced fluid disturbances. Moreover, in most biological scenarios, buoyancy and gravity forces largely counterbalance, leaving the acoustic radiation force and viscous drag force as the dominant factors governing particle motion. Under these assumptions, the acoustic radiation force, arising from sound wave scattering, serves as the principal driving mechanism for acoustophoretic manipulation. Instead, Stokes drag force, originating from nonlinear acoustic streaming, acts as a retarding force. Although the radiation force is a second-order effect, it can be expressed in terms of first-order acoustic fields as follows [29,30]:

(8) $$F_{rad}=-\pi r^{3}[\frac{2\kappa_{0}}{3}Re[f_{1}^{*}p_{1}^{*}\nabla p_{1}]-\rho_{0}Re[f_{2}^{*}v_{1}^{*}\nabla v_{1}]]$$

where $\kappa_{0}$, $f_{1}$, and $f_{2}$ are the compressibility of the fluid, monopole scattering coefficient, and dipole scattering coefficient, respectively, and the asterisk represents the complex conjugate.

The Stokes drag force due to streaming is given by:

(9) $$F_{drag}=6\pi \mu r\;(\langle v_{2}\rangle -\;v_{p})$$

where r is the particle radius and $v_{p}$ is the particle velocity. Assuming negligible inertia, Newton’s second law simplifies to $v_{p}=\langle v_{2}\rangle +F_{rad}/6\pi \mu r$. This yields an analytical expression for the particle velocity, allowing numerical prediction of particle trajectories under combined acoustic radiation and streaming forces. Since the acoustic radiation force scales with the cube of the particle radius, while the Stokes drag force is proportional to the radius squared, a critical particle size exists that delineates the transition between streaming-dominated and radiation-dominated regimes. Particles larger than this threshold tend to migrate toward pressure nodes under the influence of the radiation force.

### 2.2. Model Setup and Numerical Implementation

**Figure 1:**
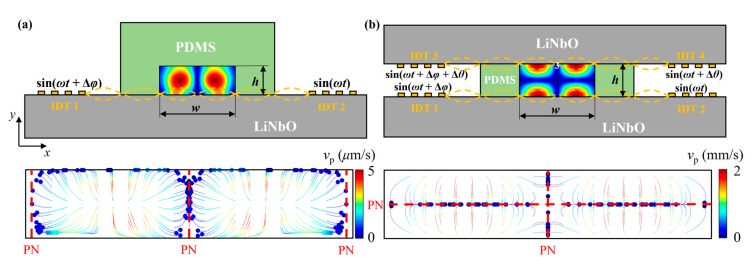
Schematic of acoustofluidic device designs and corresponding characteristic particle trajectories. (**a**) Conventional single-SSAW design. (**b**) Novel dual-sided SSAW design configured by two pairs of IDTs at the top and bottom of the microchannel.

As shown in Figure 1a, a conventional acoustofluidic device consists of a piezoelectric substrate, a pair of metallic IDTs, and microchannel walls made of highly biocompatible polydimethylsiloxane (PDMS). The width of the channel w and the height of the channel h are set to 600 μm and 125 μm, respectively. According to the channel dimensions, a resonance frequency of 6.65 MHz was selected to obtain a full wavelength within the channel width. When the IDTs are activated by electrical signals, the substrate, made of 128° Y-cut lithium niobate (LiNbO_3_), converts the signals into SAWs, propagating along the surface of the substrate. Once contacting with the bulk fluid within the microchannel, two opposing SAWs will form standing waves in the horizontal direction and radiate into the fluid at a Rayleigh angle based on the Huygens–Fresnel principle to form oscillatory traveling wave fields in the vertical direction (upper subplot of Figure 1a). If the IDTs are activated by signals with the same phase, a classical standing acoustic wave field is formed, which induces two ANs located in the middle of the left and right halves of the channel. Driven by the acoustic pressure gradient, large particles are propelled away from the antinodes, resulting in particle aggregation at PNs, as shown in the lower subplot of Figure 1a. As phase difference is prescribed between SAWs, the positions of PNs translate laterally, and thus the particle aggregation locations change accordingly. Although the traditional single-sided SSAW device can concentrate particles (Figure 1a), it introduces the acoustic energy into the channel from only one side while the opposite PDMS boundary absorbs most of the waves, significantly reducing the efficiency of particle manipulation.

Therefore, we develop a novel dual-sided SSAW device configuration featuring two LiNbO_3_ substrates on both sides of the microchannel and a pair of IDTs equipped on each substrate, as shown in Figure 1b. Utilizing this design, two SSAWs are generated along both substrates and leak into the fluid to form static standing wave fields in the vertical direction (upper subplot in Figure 1b). This dual-sided SSAW patterning strategy brings greater acoustic density within the fluid, and thus larger acoustic forces are exerted on suspended particles. Furthermore, fine-tuning the phase of four SAWs patterned on both sides will offer greater flexibility on designing particular acoustic distribution for effective particle manipulation. The representative particle trajectories of this dual-sided SSAW device in the lower subplot of Figure 1b demonstrate much faster particle movement (with maximum particle velocity of 2 mm/s compared to 5 μm/s in single-sided SSAW design) and more converged particle collection positions. While multiple collection positions are found here, we can merge them into a single collection point through dynamic phase modulation of four IDTs, which we will elaborate on and justify in the following sections.

**Figure 2:**
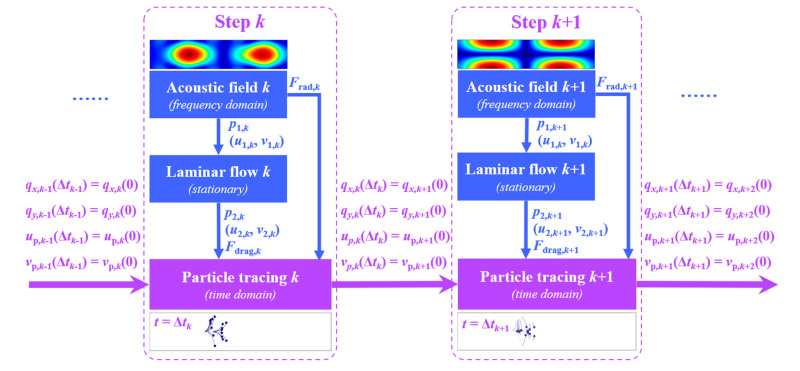
Stepwise dynamic modulation of acoustic fields, fluid flow fields, and particle fields.

To systematically study the distribution and magnitude of the first-order acoustic pressure field under dynamic phase modulation and corresponding particle movements, we established a computational model based on FEM. Assuming a uniform primary flow along the long, straight channel (streamwise *z*-direction), variations induced by acoustic waves in this direction can be neglected. To reduce computational complexity, a 2D cross-sectional model in the *x*-*y* plane is employed. Appropriate boundary conditions are imposed on the boundaries of the fluid chamber. The acoustic actuation on the surface of the substrate is prescribed through the displacement of the substrate, and thus SAW propagation on the bottom substrate can be expressed as:

(10a) $$u_{y,lower}\left(t,x\right)=0.6{u_{0}e}^{-C_{d}x}\left(\sin\left(\frac{2\pi (\frac{w}{2}-x)}{\lambda }+\omega t+\Delta \varphi \right)+sin\left(\frac{2\pi (x-\frac{w}{2})}{\lambda }+\omega t\right)\right),$$

(10b) $$u_{x,lower}\left(t,x\right)=0.6{u_{0}e}^{-C_{d}x}\left(\cos\left(\frac{2\pi (\frac{w}{2}-x)}{\lambda }+\omega t+\Delta \varphi \right)+cos\left(\frac{2\pi (x-\frac{w}{2})}{\lambda }+\omega t\right)\right),$$

where $u_{x,lower}$ denotes the transverse displacement of the bottom substrate and $u_{y,lower}$ represents the vertical displacement of the lower substrate; $u_{0}$ is the vertical displacement amplitude of the waves, $C_{d}$ is the decay coefficient in the substrates, λ is the acoustic wavelength, ω is the angular frequency of oscillating signals applied on the IDTs, and Δφ is the phase difference between the two IDTs on the same substrates. Equation (10) also stands for the displacement of the substrates in the conventional acoustofluidic device. The displacement of the upper substrate can be written as

(11a) $$u_{y,upper}\left(t,x\right)=0.6{u_{0}e}^{-C_{d}x}\left(\sin\left(\frac{2\pi (\frac{w}{2}-x)}{\lambda }+\omega t+\Delta \varphi +\Delta \theta \right)+sin\left(\frac{2\pi (x-\frac{w}{2})}{\lambda }+\omega t+\Delta \theta \right)\right),$$

(11b) $$u_{x,upper}\left(t,x\right)=0.6{u_{0}e}^{-C_{d}x}\left(\cos\left(\frac{2\pi (\frac{w}{2}-x)}{\lambda }+\omega t+\Delta \varphi +\Delta \theta \right)+cos\left(\frac{2\pi (x-\frac{w}{2})}{\lambda }+\omega t+\Delta \theta \right)\right),$$

where Δθ is the phase difference between the IDTs on the upper substrate (i.e., IDT 3 and IDT 4 in Figure 2) and the IDTs on the lower substrate (i.e., IDT 1 and IDT 2 in Figure 2). Equations (10) and (11) together describe the acoustic actuation in dual-sided SSAW device. The PDMS microchannel walls are modeled via the impedance boundary condition, governed by the following equation:

(12) $$p_{1}=Z_{n}\left(v_{1}\cdot n\right),$$

where $Z_{n}$ is the acoustic impedance of the wall and n is the normal vector of the wall.

To enable particle manipulation through a sequence of dynamically modulated acoustic fields, a customized MATLAB program is developed to track and analyze particle trajectories computed in the FEM computational package, providing reference data for phase modulation of the IDTs in the next step. Figure 2 demonstrates the computational flow of the stepwise control of acoustic fields and resultant particle trajectories. In each step, the acoustic field is firstly computed in the frequency domain through the Thermoviscous Acoustic Module. Since we have mentioned that the acoustic radiation force can be derived from the first-order results, it is calculated at this stage. Secondly, the second-order streaming flow fields are simulated in the stationary domain using the Laminar Flow Module. Here, the effects of particles on the flow fields are ignored, as the flow field is primarily driven by the introduction of SAWs. Since the acoustic frequency is in the MHz range, which is much higher than the reciprocal of the time needed for the flow to reach a steady state, the laminar flow field is analyzed for its steady-state behavior. Upon solving the second-order fields, the Stokes drag force can be obtained and utilized together with acoustic radiation force for the calculation of particle velocity and position over time via the Particle Tracing Module. Each step of particle tracing takes a duration of Δt, and the final particle positions and velocities in the current step are inherited as the initial conditions for the next step. By iteratively running the program, the continuous motion of particles controlled by varying acoustic fields is simulated. As illustrated by the representative particle trajectories in Figure 2, modulation of the acoustic fields and their corresponding nodal planes causes particles to diverge from the central line at step *k* and reconverge to it at step *k* + 1. To ensure accurate computational solutions, particularly within the thin viscous boundary layers of the flow field where the thickness is defined as $\delta =\sqrt{2\mu /\omega \rho_{0}}$, the size of the triangular mesh elements is carefully optimized. Mesh convergence is verified by progressively refining the mesh until the relative error in key parameters (e.g., first-order acoustic pressure amplitude, acoustic velocity, second-order streaming velocity) falls below 0.2% when compared to a reference solution obtained using the finest mesh. Based on this analysis, the maximum element size on the water side of the fluid–solid interface was set to 0.3 δ to adequately resolve boundary layer dynamics, while the maximum element size in the center of the water domain was set to 3 δ, ultimately yielding a total of $5\times 10^{5}$ mesh element.

## 3. Phase Influence on Acoustic Field

### 3.1. Acoustic Field Characteristics

PNs and ANs within the resultant acoustic field serve as key indicators for predicting the trajectories and final equilibrium positions of suspended particles. By modulating the phase differences Δθ and Δφ, the nodal planes of the acoustic field can be theoretically translated in the vertical and horizontal directions, respectively. To better understand the potential of phase modulation across the four IDTs and to guide the selection of appropriate acoustic fields for specific particle manipulation tasks, we conducted a comprehensive analysis of the acoustic pressure distributions under various phase configurations.

**Figure 3:**
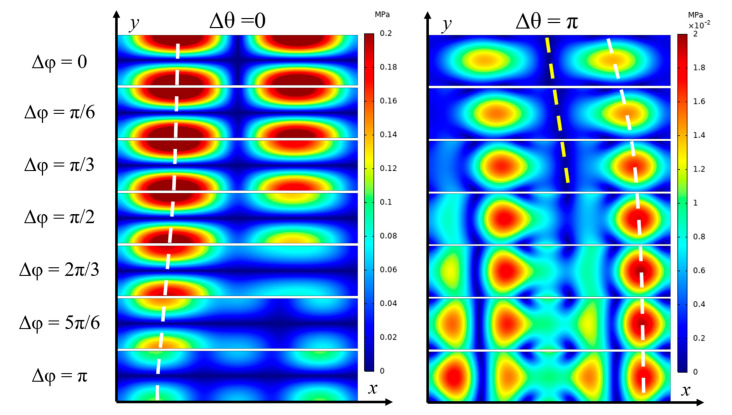
Influences of phase modulation on the first-order acoustic pressure field. The columns illustrate how varying the phase difference Δθ shifts the pressure nodal planes vertically, while the rows demonstrate that adjusting Δφ modulates the horizontal positioning of the nodal planes.

Figure 3 presents the acoustic field profiles corresponding to different SAW phase settings applied to the four IDTs. The distributions exhibit clear standing wave patterns with well-defined pressure nodal planes in both horizontal and vertical directions, where larger particles tend to be trapped. This sandwiched SSAW configuration induces acoustic resonance in both axes, resulting in a strong acoustic pressure amplitude of up to 0.2 MPa, which translates into substantial radiation forces acting on large particles. The standing wave patterns are computed under the assumptions of ideal symmetric IDT placement, synchronized phase control, and uniform boundary conditions. In practical implementations, however, deviations such as IDT misalignment, phase modulation inaccuracies, substrate inhomogeneities, and variations in the acoustic impedance of microchannel boundaries may alter the pressure field distribution, potentially impacting particle manipulation outcomes. Nonetheless, with careful device fabrication, the use of a programmable signal generator for accurate phase tuning, real-time monitoring of particle motion, and an integrated feedback control system, the desired pressure fields and corresponding particle manipulation can still be reliably achieved.

In our study, a series of acoustic fields were generated to drive large particle motion. Phase differences Δθ between the top and bottom SSAWs primarily shift the horizontal nodal planes vertically. Given that our dual-sided SSAW system rapidly drives particles toward the horizontal nodal plane (as shown in Figure 1), further vertical adjustment is generally unnecessary. Therefore, we focus on Δθ=0 and Δθ=π for comparative analysis, which are presented in the left and right columns of Figure 3, respectively. Conversely, phase differences Δφ between the two IDTs on the same substrate shift the vertical nodal planes horizontally. In our device, particle aggregation results in two trapping locations within a single wavelength, as shown in Figure 3. By dynamically tuning Δφ from −π to π in finer steps of π/6, it is possible to steer particles from one cluster toward the other, ultimately converging them into a single focal point. Through modulation of both Δθ and Δφ, two-dimensional particle control is achieved. Furthermore, incorporating an additional set of IDTs in the streamwise direction would enable straightforward extension to full three-dimensional manipulation.

As shown in Figure 3, for the series of acoustic fields with Δθ=π, the vertical nodal plane shifts rightwards as Δφ varies from 0 to π/3. The change in the position of vertical PN Δd under a phase change of Δφ can be determined theoretically as:

(13) Δd=Δφ/2k,

where *k* is the wave number. The numerical results indicate that the vertical nodal plane shifts approximately 25 μm as Δφ changes by π/6, which aligns well with theoretical analysis. The yellow dashed line illustrates the linear shift in the vertical nodal plane as Δφ increases from 0 to π/3. However, as Δφ further increases, the linear relationship disappeared due to the influence of the channel sidewalls.

Due to the confined channel dimensions, wave reflection from the channel boundaries restricts further lateral displacement of the nodal and antinodal planes. This limitation prevents antinodes from moving closer to the channel walls. As illustrated by the white dashed line in Figure 3, the ANs gradually approach the sidewall as Δφ increases, but their movement is restricted, eventually halting at approximately 50 μm from the sidewall. A similar trend is observed in the group of acoustic fields with Δθ=0, also indicated by the dashed white line. Based on the observed phase-induced shifts in the acoustic field, a potential strategy emerges: first use the acoustic field with Δθ=0 to concentrate particles along the horizontal nodal plane, and then induce lateral movement of particles along that plane using Δθ=π and Δφ within the range of (−π/3,π/3).

### 3.2. Phase Modulation Design for Particle Manipulation

While our dual-sided SSAW device without phase modulation effectively concentrates particles along the horizontal nodal plane, it results in the formation of multiple discrete aggregation sites rather than a single collection point. To enhance the spatial resolution of particle aggregation within the microchannel, it is necessary to generate stronger lateral forces on the particles. Given the significant pressure gradients surrounding the ANs (highlighted in red in Figure 3), particles are rapidly repelled from these regions. Therefore, by dynamically modulating the positions of the ANs through phase control, we can generate spatially localized acoustic forces that enable continuous lateral movement of particles along the pressure nodal plane, offering a promising approach for precise and controllable particle positioning. Effective manipulation in the horizontal direction requires careful selection of several key parameters, including not only the distance *d* between the position of the targeted particle cluster $\left(x_{p,0},\;y_{p,0}\right)$ and the position of the AN in the imposed acoustic field, but also the time interval of each phase modulation step.

**Figure 4:**
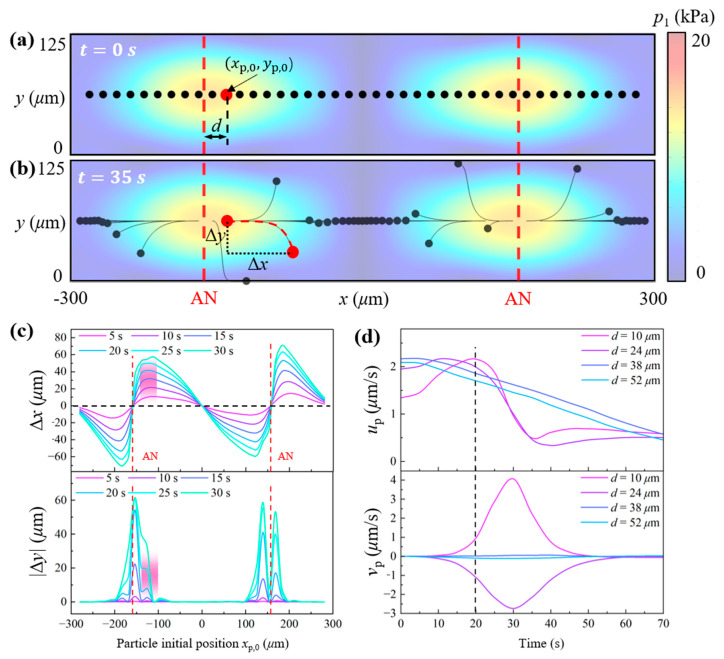
Influence of the relative distance between particle position and AN position on particle movement. (**a**) Initial particle positions; (**b**) particle trajectories after 35 s of acoustic stimulation; (**c**) particle displacement along the *x*- and *y*-axes; (**d**) particle velocity components in the *x*- and *y*-directions.

In this section, the influences of these two key parameters (i.e., distance *d* and time *t*) on particle motions are examined using a group of particles evenly distributed on the horizontal nodal plane of a representative acoustic field with Δθ=π, Δφ=0, and $u_{0}=0.1$ nm as shown in Figure 4a. The particle radius is set to 5 μm, which is above the critical size threshold for our case [9]. Our analysis holds valid for microparticle sizes beyond this critical value, where particle motion is predominately governed by the acoustic radiation force. Suggested values of these key parameters are empirically acquired to balance the lateral propelling distance and the unwanted particle escape from the horizontal nodal plane. Since the acoustic field is horizontally symmetric, only the rightward propelling effects in the left half channel are analyzed. As shown in Figure 4, ANs and PNs in the horizontal direction are positioned at x=±150 μm and x=0, ±300 μm, respectively, while in the vertical direction, PNs and ANs are featured at y=0, 120 μm and y=60 μm. The *x*-axis and *y*-axis displacement of particles with different initial positions is presented in Figure 4c. As the strong pressure gradient near ANs, particle displacements along both axes are predominant near ANs, showcased by the peaks in Figure 4c. The optimal range of AN positions relative to the particle position, where maximized lateral displacement and minimal vertical displacement are achieved, is highlighted by pink bands (10 μm≤d≤50 μm). While particles with larger values of *d* exhibit negligible *y*-axis displacement, their corresponding *x*-axis displacement is also relatively small. This illustrates a trade-off between minimizing undesired vertical motion and maximizing effective lateral transport, which must be carefully balanced during acoustic field design for precise particle manipulation. Thus, in particle manipulation practices in the following sections, d=30 μm is adopted.

Although the acoustic field designed based on the ideal AN position range can initially drive particles laterally with minimal displacement along the *y*-axis, noticeable *y*-axis displacement tends to occur after a certain period of acoustic stimulation. Therefore, it is essential to determine the specific time window during which particles can be reliably maintained on the horizontal pressure nodal plane. Four particles, whose initial positions relative to the ANs are among the optimal ranges to induce significant *x*-direction displacement, are picked to investigate the variation in their velocity components in the *x*- and *y*-directions over time, as illustrated in Figure 4d. The *x*-direction components of particle velocity with d=38 and 52 μm decrease gradually and smoothly over time, while for particles with d=10 and 26 μm, their *x*-axis velocities drop dramatically from 20 s. This is because the particles with d=10 and 26 μm start to deviate vertically after approximately 20 s (as shown in Figure 4c), which in turn weakens their *x*-component velocities. To maintain effective lateral transport, a new acoustic field, configured such that the updated distance between the particle position and the antinode satisfies d=30 μm, should be introduced via phase modulation at this point. Through stepwise phase modulation of the acoustic field, particles experience sustained lateral propulsion with relatively high speed and directional control. For the initial distance of *d* = 30 μm, a phase modulation time step of 20 s is selected to prevent particles from significantly diverging from the nodal plane and retain relatively high *x*-direction speed.

## 4. Particle Manipulation

### 4.1. Single Particle Manipulation

**Figure 5:**
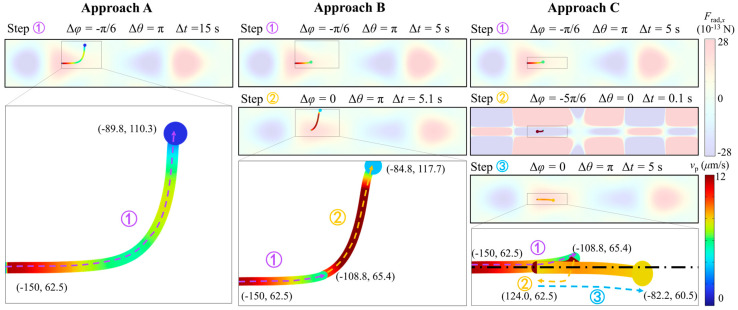
Single particle maneuvering along horizontal nodal plane driven by different phase modulation strategies.

In this section, a single particle with a radius of 5 μm, positioned on the horizontal pressure nodal plane, is manipulated using three different phase modulation strategies to compare their effectiveness in driving lateral particle movement, as shown in Figure 5. A displacement amplitude of 0.2 nm is selected to more accurately reflect experimentally relevant conditions. A particle is initially positioned at x=−150 μm, and an acoustic field with an antinode located at x=−175 μm is selected based on the conclusions drawn in Section 3.2. The corresponding *x*-direction radiation force field is also illustrated in the background for reference. As shown in the left column of Figure 5, approach A does not involve dynamic phase modulation and relies on a single, static acoustic field to drive particles towards the pressure node. While this method is straightforward to implement, it exhibits limitations in both spatial precision and temporal efficiency. Although the particle initially attains a high velocity, it begins to deviate from the horizontal nodal plane and significantly slows down after a short period of time. Thus, additional phase modulation is required to maintain the particle’s trajectory along the horizontal nodal plane and prevent vertical divergence.

Following up the dynamic phase modulation strategy proposed in Section 3.2, approach B demonstrates that shifting the acoustic field using solely Δφ to maintain an optimal distance between the particle and the pressure node can significantly accelerate lateral manipulation, as shown in the middle column of Figure 5. Compared to approach A, approach B, using two sequential acoustic fields, achieves a greater *x*-direction displacement in only two-thirds of the time required by approach A. However, it also introduces a *y*-direction deviation of approximately 50 μm, which complicates further manipulation steps. These vertical drifts arise from the *x*-direction radiation force $F_{rad,x}$ which is primarily concentrated along the horizontal nodal line of the channel and diminishes sharply away from it. Therefore, while approach B achieves remarkable lateral displacement, it is not sustainable for long-distance particle transport due to loss of confinement along the nodal plane.

Approach C, enabled by the special design of the dual-SSAW microfluidic device, offers a phase modulation solution that relies on both Δθ and Δφ to manipulate particles in both the *x*-direction and *y*-direction, as illustrated by the right column of Figure 5. Specifically, in the first step of approach C, the same acoustic field used in approaches A and B is selected and activated for 5 s. Since the first step induces slight vertical deviation of particle movement, an acoustic field with Δθ=0 is applied in the second step to pull the particle vertically back onto the central pressure nodal plane. Although this step may appear to reduce the net lateral displacement, it effectively corrects the particle trajectory and requires only 0.1 s to complete. In the third step, a new acoustic field is applied based on the updated particle position by adjusting Δφ. Similar to the strategy used in approach B, this allows the particle to be further propelled in the lateral direction. As a result, although the second step sacrifices a small amount of *x*-direction displacement, approach C achieves a greater overall lateral displacement within the same time frame as approach B, while simultaneously maintaining the particle on the pressure nodal plane. Therefore, iteratively applying approach C offers an efficient strategy for shuttling particles over long lateral distances. This is adopted in the following section for multi-particle concentration applications.

### 4.2. Multiple Particles

**Figure 6:**
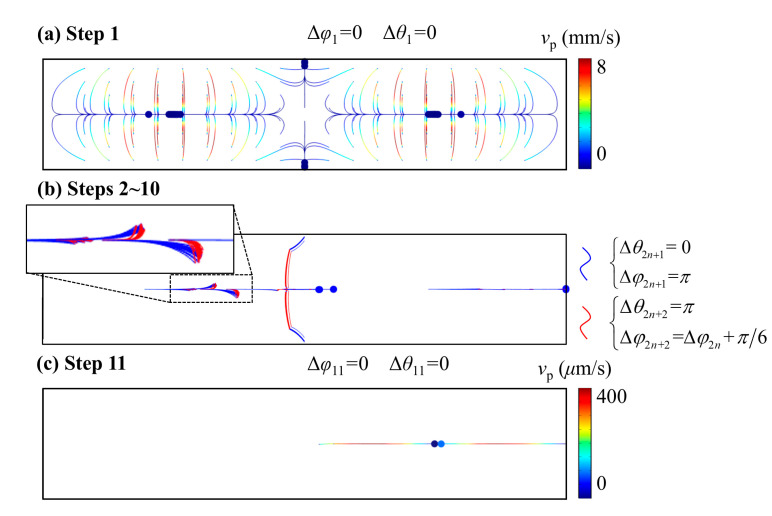
Stepwise particle manipulation strategy by a sequence of 11 phase-modulated acoustic fields.

Microparticle aggregation is a classical task in the field of acoustofluidics. To further improve aggregation efficiency and increase the collection rate, our stepwise phase modulation method, based on approach C proposed in Section 4.1, enables the rapid concentration of nearly all particles to a single focal point. As shown in Figure 6a, particles that are initially uniformly distributed across the cross-section of the microchannel are collected at multiple locations. After sequentially applying 11 phase-modulated acoustic fields, as depicted in Figure 6c, all particles are successfully concentrated at the middle of the right half of the channel. This highly focused aggregation is promising for the downstream particle collection and processing. To address these issues, our stepwise method enables the concentration of almost all particles to a single point. As shown in Figure 6a, the particles are initially uniformly distributed across the cross-section of the microchannel. After sequentially applying 11 acoustic fields, as depicted in Figure 6c, all particles are concentrated at the middle of the right half of the channel. This concentrated result is promising for the subsequent collection of the particles.

To further explain the mechanism and details of each step in our approach, the entire procedure is divided into three stages. First, as shown in Figure 6a, an acoustic field with Δθ=0, Δφ=0 is applied to initially concentrate the particles at four local PNs. The goal is to guide all particles toward the PN located at the right side of the channel. Therefore, a series of acoustic fields are subsequentially applied to propel the particles laterally, as shown in Figure 6b. In this stage, the iterative application of approach C from Section 4.1 is employed. The zoomed-in plot in Figure 6b shows particle trajectories identical to those in approach C (Figure 5), where back-and-forth movement is employed to maintain particle alignment along the horizontal nodal plane and suppress vertical deviation. Eventually, once all particles are transported to the right half of the channel, the acoustic field with Δθ=0, Δφ=0 is re-applied. At this point, since the particles remain aligned along the central pressure nodal plane, they migrate further rightwards and ultimately aggregate at the rightmost node.

**Figure 7:**
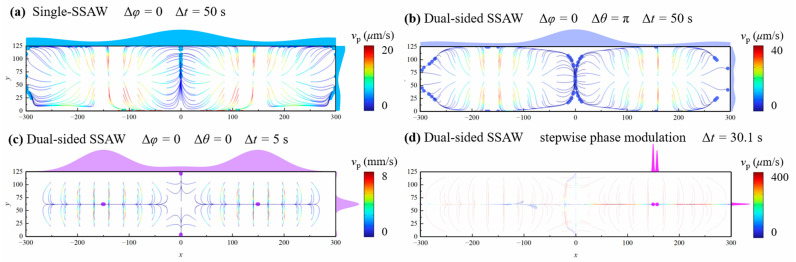
Particle trajectories and final particle distribution under different SSAW configurations and phase modulation strategies.

Figure 7 compares the particle aggregation performance of four different schemes, each employing distinct device setups and phase modulation strategies. In all cases, particle trajectories are simulated under the same SAW amplitude of $u_{0}=0.2$ nm. As shown in Figure 7a, the traditional single-SSAW setup introduces acoustic energy from only the bottom side of the channel, resulting in slower particle manipulation compared to the other schemes. In addition to its low efficiency, the final particle distribution is not satisfactory, as particles remain dispersedly distributed along the pressure nodal plane without forming distinct concentration points. Similarly, the dual-sided SSAW method with phase modulation of Δθ=π, Δφ=0, as shown in Figure 7b, also yields an unsatisfactory concentration outcome, despite the increased particle velocity. The fastest particle aggregation is achieved using the dual-sided SSAW system with a phase setting of Δθ=0, Δφ=0, as presented in Figure 7c. Under this configuration, all particles are concentrated at four nodal points within 5 s, which demonstrates great enhancement in spatiotemporal aggregation efficiency. However, the presence of multiple concentration locations poses challenges for collecting particles from the main fluid stream. Build upon this method, our proposed method, utilizing a dual-sided SSAW system combined with a dynamic phase modulation strategy (approach C, as described in Section 4.1), drives all particles to a single focal point within approximately 30 s (Figure 7d). This method greatly improves spatial resolution of particle aggregation and demonstrates great potential for achieving high collection rate within an acceptable time frame, making it well-suited for practical application.

## 5. Conclusions

In this study, we proposed a novel phase-modulated, dual-sided SSAW microfluidic device, incorporating four independently controlled IDTs. This configuration allows four SAWs to be coupled when being emitted into the liquid chamber from its four corners, forming two SSAWs at the top and bottom boundaries of the microchannel. By dynamically tuning the phase of the four SAWs, desirable acoustofluidic fields can be produced to efficiently move microparticles. To systematically investigate the performance of this design, we established a 2D cross-sectional FEM model, so as to study the influence of phase modulation on the resulting acoustic field. Both the horizontal and vertical shifts in the pressure nodal planes could be achieved through the dynamic phase modulation, offering great potential for flexible particle manipulation. Particles under the influence of the dual-sided SSAW field, in the absence of phase modulation, were seen to undergo significant vertical displacements, resulting in rapid aggregation to the horizontal nodal planes. However, relatively slow lateral displacements over these horizontal nodal planes and the formation of multiple aggregation regions limit the efficiency and spatial resolution of the particle enrichment. As a foundation for designing effective phase-modulated acoustic fields to improve the particles’ lateral movement, the response of a single particle was first studied under a representative modulation condition. This analysis confirms that a distance of 30 μm between the particle position and the location of the AN in the designed acoustic field and a SAW duration of 20 s yielded maximized lateral displacement and minimized unintended deviations.

To achieve aggregation at a single collection location within the bulk fluid, various dynamic phase modulation strategies were proposed and evaluated through stepwise particle trajectory simulations. Among these, a strategy involving iterative back-and-forth displacement of particles was adopted for multi-particle aggregation. This approach induced particles to move rapidly along the horizontal nodal plane from one side to the other, ultimately converging them to a single focal point within approximately 30 s. Compared to conventional designs, this novel method demonstrates significantly improved spatial resolution and aggregation efficiency, offering significant advantages for particle manipulation.

## Data Availability

The data that support the findings of this study are available from the corresponding author upon reasonable request.
